# ECT-Resistant Bipolar Depression Treated with a Single Course of Triple Chronotherapy

**DOI:** 10.1155/2022/9957190

**Published:** 2022-02-14

**Authors:** Jadon Webb

**Affiliations:** Bloom Mental Health LLC, Yale Child Study Center, USA

## Abstract

The combination of sleep deprivation, sleep phase advance, and bright light is termed triple chronotherapy (TCT). TCT is a promising treatment for depression, but it is not clear how effective it is for depression resistant to conventional treatments such as medications. Here, we report a case of triple chronotherapy effectively treating depression resistant to both medication and an extended course of bilateral electroconvulsive therapy. Triple chronotherapy may be a useful method to consider in cases of severe, treatment-resistant depression.

## 1. Introduction

No consensus definition exists for treatment-resistant depression (TRD) [1]; however, many investigators classify TRD as lack of response to an adequate trial of at least two treatments, typically oral medications. The odds of patients responding to oral medications decrease precipitously with each failed trial. These patients are thus often advised to start looking beyond oral medication and consider modalities such as intravenous ketamine, transcranial magnetic stimulation (TMS), and electroconvulsive therapy (ECT).

ECT is more effective for depression than oral antidepressants and has generally been regarded as a gold standard for the most difficult to treat cases. Bilateral ECT (stimulus applied to both sides of the brain) is considered the most effective ECT protocol [2], although memory loss can be significant. Despite this, there are cases of TRD that do not respond to bilateral ECT or other intensive conventional treatments. In these cases, clinicians can sometimes be at a loss for what to offer next.

Some studies [3], although not all [4], have suggested that triple chronotherapy (TCT) may be a safe, effective, and rapid treatment for mood disorders, including both unipolar and bipolar depression. Those who respond to treatment usually do so within a few days and can show sustained improvement well beyond the initial night of sleep deprivation. TCT has been successfully used for severe depression, including with suicidal ideation [5]. Early data also suggests that TCT may be beneficial for treating TRD [5, 6].

If indeed TCT does prove to be an effective treatment for TRD, it would represent a major advancement by offering patients a safe and unique way to help this difficult disorder. The case I report here illustrates this: a patient successfully treated with TCT who had previously failed numerous oral medications and multiple courses of bilateral ECT.

## 2. Case Presentation

### 2.1. Patient Description

The patient is a 23-year-old male who presented for severe depression (Quick Inventory of Depressive Symptomatology-Self-Report [7] score of 22). His depressive symptoms were characterized by deep sadness, anhedonia, flat affect, exhaustion, excess sleep, hopelessness, intermittent auditory and visual hallucinations, and suicidal ideation, for which he had been hospitalized in the past. Due to the severity of symptoms, he dropped out of school and was unemployed, spending most days alone in his room. He would periodically sit motionless for hours, minimally responsive to stimulation, and appearing to be “frozen.” The clinical team suspected intermittent catatonia.

Bipolar disorder was diagnosed as he experienced intermittent periods of increased risk taking and goal directed activity that were a marked departure from his usual functioning and lasted for multiple days. The patient was additionally diagnosed with narcolepsy, confirmed by polysomnography. He experienced extreme fatigue most days, with a strong preference to stay up late at night.

### 2.2. Prior Treatments

Multiple antidepressants, mood stabilizers, antipsychotics, stimulants, and psychotherapy had been tried for years with minimal improvement. Fifty sessions of ECT (both unilateral and bilateral) were administered as a continuous series over a period of 24 months. The treatment protocol began as unilateral ECT but was switched to bilateral due to inadequate treatment response. Despite this switch, ECT was still minimally effective for the depression symptoms, and it caused disabling memory impairment. ECT treatment was thus terminated, and the patient continued to suffer from debilitating depressive symptoms.

### 2.3. TCT Protocol

**Figure 1:**
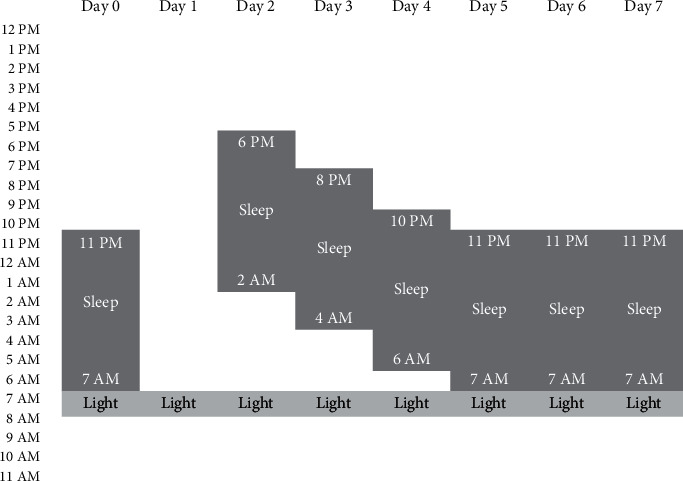
Triple chronotherapy protocol used in this case. *x*-axis shows the days of the protocol; *y*-axis is time of day. A night of total sleep deprivation (day 1) is followed by phase advanced reintroduction of sleep (patient asleep at 6 PM following sleep deprivation, advancing the next night to 8 PM, and so on). 30 minutes of bright light was administered each morning at 7 AM.

I prescribed a course of TCT (Figure 1), with a night of total sleep deprivation at home, followed by sleep phase advance, with no changes in medication. Each morning, the patient was exposed to 2,500 lux for 30 minutes with a consumer-grade light box. He was permitted to drink caffeine in moderation during treatment and instructed to avoid napping or oversleeping. Family members helped the patient stay awake and adhere to the protocol, and the patient regularly followed up in our clinic. Since the patient had no recent history of manic symptoms and had poor response to numerous mood stabilizers, he was continued on fluoxetine 20 mg daily with close observation for any emergent manic symptoms.

### 2.4. TCT Results

The patient reported extreme exhaustion during the first 3 days of the protocol; however, he noticed profound changes in mood by the third day, stating he was “exhausted but not sad.” He experienced no other significant side effects from the treatment. Depression rating scale scores (QIDS) decreased from 22 (i.e., very severe depression) to 5 (no depression) within 5 days, and this was sustained in subsequent clinical visits. The patient experienced no manic symptoms throughout the protocol and in the several years of follow-up observation.

Objectively, the patient appeared dramatically different on exam the week following the protocol. His affect was bright and full, and his prior feelings of sadness, anhedonia, and suicidal thoughts had fully remitted. Family who attended follow-up clinical visits noted the remarkable change, stating that they “had their family member back” for the first time after years of debilitating symptoms. These clinical benefits were significant enough that he was able to restart a career in sports and begin rebuilding his social network.

The patient described feeling as though he had awoken from a “depression coma” lasting for much of his early adulthood. I have followed up with this patient for several years after this initial described TCT treatment, and he has since experienced several depression symptom relapses corresponding to seasonal change (worse in the winter) and also to times when he began napping during the day or oversleeping in the morning. Repeating the TCT protocol has been able to treat these depressive symptoms back to remission.

## 3. Discussion

Depression is a complex illness that is notoriously difficult to treat to remission. Traditional antidepressants have limited efficacy [8] and can take weeks to begin working. Adjunctive treatments such as electroconvulsive therapy are effective but also costly and time-consuming and can have undesirable side effects.

Triple chronotherapy has a well-established record of safety, and some studies suggest it may be very effective in certain populations, perhaps superior to oral antidepressants in some cases. In our clinic, I have observed multiple instances of TCT being effective for treatment-resistant depression but was nonetheless surprised at how effective it was in treating this extreme case, resistant also to bilateral ECT.

In addition to being highly effective, TCT offers a drug-free treatment option that appeals to some of our patients that are hesitant to try pharmaceuticals. By no means does TCT replace well-established pharmaceutical treatments for all patients, and indeed, TCT often works well when paired with medication treatment. Nonetheless, I find that many patients greatly appreciate this creative and alternative approach to treating depression.

## Data Availability

Not applicable: all relevant data is contained in the manuscript.
